# Ten-year retrospective analysis of multiple trauma complicated by pulmonary contusion

**DOI:** 10.1186/2054-9369-1-7

**Published:** 2014-05-01

**Authors:** Hui Jin, Li-Qun Tang, Zhi-Guo Pan, Na Peng, Qiang Wen, You-Qing Tang, Lei Su

**Affiliations:** Intensive Care Unit, General Hospital of Guangzhou Command, Guangzhou, 510000 China

**Keywords:** Multiple trauma, Pulmonary disease, Retrospective studies

## Abstract

**Background:**

This study reports a 10-year retrospective analysis of multiple trauma complicated by pulmonary contusion. The purpose of this study is to ascertain the risk factors for mortality due to trauma in patients with pulmonary contusion, the impact of various treatment options for prognosis, and the risk factors for concurrent Acute Respiratory Distress Syndrome (ARDS).

**Methods:**

We retrospectively analyzed 252 trauma patients with lung contusion admitted to the General Hospital of Guangzhou Command from January 2000 to June 2011 by using the statistical processing system SPSS 17.0 for Windows.

**Results:**

We included 252 patients in our study, including 214 males and 38 females. The average age was 37.1 ± 14.9 years. There were 110 cases admitted to the ICU, of which 26 cases with ARDS. Nine of the 252 patients died. We compared those who survived with those who died by gender and age, the difference was not statistically significant (*P* = 0.199, *P* = 0.200). Separate univariate analysis of those who died and those who survived found that shock on admission (*P* = 0.000), coagulation disorders (*P* = 0.000), gastrointestinal bleeding (*P* = 0.02), the need for emergency surgery on admission (*P* = 0.000), pre-hospital intubation (*P* = 0.000), blood transfusion within 24 hours (*P* = 0.006), the use of mechanical ventilation (*P* = 0.000), and concurrent ARDS (*P* = 0.000) are poor prognosis risk factors. Further logistic analysis, including the admission GCS score (*OR* = 0.708, 95% CI 0.516-0.971, *P* = 0.032), ISS score (*OR* 1.135, 95% CI 1.006-1.280, *P* = 0.039), and concurrent ARDS (*OR* = 15.814, 95% CI 1.819-137.480, *P* = 0.012), identified the GCS score, ISS score and concurrent ARDS as independent risk factors of poor prognosis. Shock (*OR* = 9.121, 95% CI 0.857-97.060, *P* = 0.067) was also related to poor prognosis. Patients with injury factors such as road accident, falling injury, blunt injury and crush injury, et al.(*P* = 0.039), infection (*P* = 0.005), shock (*P* = 0.004), coagulation disorders (*P* = 0.006), emergency surgery (*P* = 0.01), pre-hospital intubation (*P* = 0.000), chest tube insertion (*P* = 0.004), blood transfusion (*P* = 0.000), usage of hormones (*P* = 0.002), phlegm (*P* = 0.000), ventilation (*P* = 0.000) were at a significantly increased risk for ARDS complications.

**Conclusions:**

Those patients with multiple trauma and pulmonary contusion admitted to the hospital with shock, coagulopathy, a need for emergency surgery, pre-hospital intubation, and a need for mechanical ventilation could have a significantly increased risk of mortality and ARDS incidence. A risk for poor prognosis was associated with gastrointestinal bleeding. A high ISS score, high APACHE2, and low GCS score were independent risk factors for poor prognosis. If patients developed an infection or were given drainage, hormones, and phlegm treatment, they were at higher risk of ARDS. Pre-hospital intubation and drainage were independent risk factors for ARDS. In patients with ARDS, the ICU stay, total length of stay, and hospital costs might increase significantly. A GCS score < 5.5, APACHE 2 score > 16.5, and ISS score > 20.5 could be considered indicators of poor prognosis for patients with multiple trauma and lung contusion.

## Background

In the United States, the overall fatality rate of multiple injuries was approximately 12%, the fatality ranking the fourth in the United States. Multiple trauma accounted for 16% of the world's medical expenses [1]. In 2004, an American study found that the hospitalization costs for trauma patients with ARDS were far higher than the costs for patients without ARDS complications [2]. Chest injury is one of the most common multiple injuries. Approximately one-third of multiple trauma patients experience the complication of chest contusion [3], which is prone to cause subsequent lung contusion. A study from America reviewed 6332 patients with multiple trauma whose ISS scores were greater than 15 points. Of these, 1722 (27%) patients had lung contusion [4], and the overall mortality rate was 10%-25% [5]. In China, lung contusion accounts for approximately 5% of the trauma incidence, and it is an independent risk factor for ALI, ARDS, and VAP [6]. The mortality rate of lung contusion was as high as 14%-40% [7].

Our hospital is the largest trauma care center in south China. In this study, we retrospectively analyzed and summarized patients admitted to our hospital from January 2000 to June 2011 who experienced lung contusion after trauma to clarify the risk factors for trauma mortality in patients with pulmonary contusion, to determine the impact of various treatment options on prognosis, and to identify risk factors for the development of concurrent ARDS. Risk factors for mortality associated with lung contusion in patients with multiple injuries in south China were statistically analyzed to evaluate the value of the GCS, ISS, and APACHE II scores on predicting prognosis and ARDS and to research the related economic impact of lung contusion.

## Methods

### Source of data

Patients diagnosed as “multiple trauma complicated with pulmonary contusion” who were admitted to the General Hospital of Guangzhou Command from January 2000 to June 2011 were included in this study.

### Case inclusion criteria

Patients were included in this study if they: 1. Met the diagnostic criteria for multiple injuries: serious damage to two or more anatomical sites, and life threatening-injuries when each injury is considered independently; 2. Experienced pulmonary contusion (patch or large shadows on X-ray/CT scan, the edge of shadows is unclear. We used CT Volume Index (CTVI) to evaluate the severity of pulmonary contusion. CTVI score was calculated based on the ratio of affected lung to total lung [slices of lung on CT × affected pixel region/lung pixel region × 0.45 (left side) + slices of lung on CT × affected pixel region/lung pixel region × 0.55(right side)].) [8]; 3. Adults over 18 years old; 4. Admitted within 24 hours after injury and hospitalized for more than 48 hours.

### Case exclusion criteria

Patients were excluded from this study if they: 1. Underwent long-term treatment (more than one week) outside the hospital with poor efficacy; 2. Had a non-trauma-induced pulmonary contusion; 3. Exhibited serious disease or organ dysfunction prior to injury; 4. Dropped out of treatment; 5. Incomplete cases.

### Observed

Patients’ general data, including sex, age, injury factors (car accident, fall injuries, crushing, heavy injury. or other), the site of injury (combined head injury, combined abdominal injury), and complications (shock, coagulation disorders, liver and kidney dysfunction, gastrointestinal bleeding) were collected in this study. The GCS, APACHE2, and ISS scores were used to evaluate the patient's admission consciousness, severity of disease. and injury assessment. Each patient’s treatment, prognosis, and health economic indicators were statistically analyzed and included the need for emergency surgery, pre-hospital intubation, hormones, pain, bleeding, suctioning, ventilator and antibiotics, survival, the total length of stay, ICU length of stay, and hospital costs.

### Statistical analysis

The SPSS17.0 software was used to create a database and to perform statistical analyses. If the measurement data met the normal distribution, the groups were compared by *t*-test; if they did not meet the normal distribution, non-parametric tests were used to compare the variables. Count data were compared between groups using the chi-square test and Fisher's exact test. Risk factors and related factors were analyzed using logistic regression analysis. A level of *P* = 0.05, *P* < 0.05 was considered statistically significant.

## Results

### General Information

A retrospective analysis of nearly 10 years of clinical cases was performed. There were a total of 1,134 cases of multiple trauma. We collected 397 cases that were diagnosed as multiple injuries combined with lung contusion; 137 cases were excluded due to the application of the exclusion criteria, and 8 cases died within 24 hours of admission. Therefore, a total of 252 patients were included in our study, including 214 males and 38 females. The average age was 37.1 ± 14.9 years. There were 110 cases were admitted to the ICU, of which 26 cases with ARDS. Of the included cases, 9 patients died, and 243 survived. The survival group was compared with those who died by gender and age; the difference was not statistically significant (*P* = 0.199, *P* = 0.200).

### Analysis of risk factors for death

#### Univariate analysis

A separate univariate analysis was conducted for the group of those who died and for the group of those who survived (N = 252) (Table 1). This analysis showed that shock on admission (*P* = 0.000), coagulation disorders (*P* = 0.000), gastrointestinal bleeding (*P* = 0.02), the need for emergency surgery on admission (*P* = 0.000), pre-hospital intubation (*P* = 0.000), blood transfusion within 24 hours (*P* = 0.006), the use of mechanical ventilation (*P* = 0.000), and concurrent ARDS (*P* = 0.000) are risk factors for poor prognosis. The GCS score of those who died was 5.13 ± 4.09, which was significantly lower than that of the survival group (11.44 ± 4.19, *P* = 0.000). The APACHE2 score was 25.13 ± 7.47, and the ISS score was 33.88 ± 10.56 for the group of patients who died, both of which were significantly higher than the survival group (16.29 ± 7.96, *P* = 0.000; 20.49 ± 9.69, *P* = 0.000). These results suggest that in combination with the GCS score, the APACHE2 score and the ISS score can be used to determine the prognosis of patients.

**Table 1:** **Univariate analysis of the prognosis of patients with pulmonary contusion**

Items	Death group	Survival group	***P-***value
Injury factors			
Road accident	8	163	0.602
Falling injury	0	42	
Blunt injury	0	14	
Crush injury	0	12	
Others	1	12	
Concurrent situation			
Hemothorax	2	41	0.543
Pneumothorax	1	15	
Hemopneumothorax	3	46	
Rib fractures	1	100	
Combined head injury	2	46	0.104
Abdominal injury	2	13	
Damage to other parts	4	72	
Infection	0	16	0.427
Shock	4	17	0.000
Coagulation disorders	4	18	0.000
Liver dysfunction	1	16	0.596
Renal dysfunction	2	15	0.06
Gastrointestinal bleeding	1	3	0.02
GCS	5.13 ± 4.09	11.44 ± 4.19	0.000
APACHE 2	25.13 ± 7.47	16.29 ± 7.96	0.000
ISS	33.88 ± 10.56	20.49 ± 9.69	0.000
Emergency surgery	6	36	0.000
Pre-hospital intubation	7	41	0.000
Chest tube insertion	3	71	0.79
Blood transfusion	7	80	0.006
Usage of hormone	4	58	0.16
Pain relief	2	109	0.18
Reversal of anticoagulation	6	164	0.959
Suctioning	7	144	0.267
Mechanical ventilation	7	29	0.000
Concurrent ARDS	6/9	20/243	0.000

#### Logistic regression analysis

A logistic analysis of further results showed that the variance indicated by the regression equation, including admission GCS score (*OR* = 0.708, 95% CI 0.516-0.971, *P* = 0.032), ISS score (*OR* = 1.135, 95% CI 1.006-1.280, *P* = 0.039), and concurrent ARDS (*OR* = 15.814, 95% CI 1.819-137.480, *P* = 0.012), identified the GCS score, ISS score, and concurrent ARDS as independent risk factors of poor prognosis. Shock (*OR* = 9.121, 95% CI 0.857-97.060, *P* = 0.067) was also related to poor prognosis (death).

### Analysis of concurrent ARDS risk factors

#### Univariate analysis

As shown in the following table (Table 2), patients with injury factors (*P* = 0.039), infection (*P* = 0.005), shock (*P* = 0.004), coagulation disorders (*P* = 0.006), emergency surgery (*P* = 0.010), pre-hospital intubation (*P* = 0.000), chest tube insertion (*P* = 0.004), blood transfusion (*P* = 0.000), usage of hormones (*P* = 0.002), suctioning (*P* = 0.000), and mechanical ventilation (*P* = 0.000) have a significantly increased risk of ARDS complications.

**Table 2:** **Univariate analysis of multiple trauma with pulmonary contusion patients with ARDS**

Items	ARDS group	Non-ARDS groups	***P***-value
Gender (male)	23	191	0.595
Age	34.5 ± 11.8	34.9 ± 13.8	0.908
Injury factors			
Road accident	24	147	0.039
Falling injury	1	41	
Blunt injury	0	14	
Crush injury	0	12	
Other	1	12	
Concurrent situation			
Hemothorax	3	19	0.218
Pneumothorax	0	16	
Hemopneumothorax	12	75	
Rib fractures	16	88	
Combined head injury	7	41	0.064
Abdominal injury	2	13	
Damage to other parts	11	65	
Infection	5	11	0.005
Shock	6	15	0.004
Coagulation disorders	6	16	0.006
Liver dysfunction	2	15	0.839
Renal dysfunction	3	14	0.305
Gastrointestinal bleeding	1	3	0.331
GCS	9.31 ± 4.97	11.5 ± 4.21	0.000
APACHE 2	20.27 ± 8.61	15.88 ± 7.86	0.000
ISS	22.46 ± 9.47	21.19 ± 10.64	0.000
Emergency surgery	9	33	0.01
Prehospital intubation	19	29	0.000
Drainage	14	60	0.004
Blood transfusion	18	69	0.000
Antibiotic use	26	226	0.000
Hormone	13	49	0.002
Pain relief	13	98	0.519
Hemostatic	20	150	0.278
Suctioning	24	127	0.000
Mechanical ventilation	26	10	0.000
ICU stay	28.31 ± 51.01	8.43 ± 7.93	0.000
Total length of stay	56.85 ± 59.81	43.95 ± 45.30	0.022
Hospitalization costs	152816.46 ± 144291.31	66635.67 ± 53894.77	0.000

### Logistic regression analysis

A logistic regression analysis of further results showed that the *OR* of ARDS complications was significantly related to pre-hospital intubation (*OR* = 18.633, 95% CI 7.01-49.528, *P* = 0.000) and drainage (*OR* = 2.911, 95% CI 1.116-7.952, *P* = 0.029).

### ROC curve

**Figure 1:**
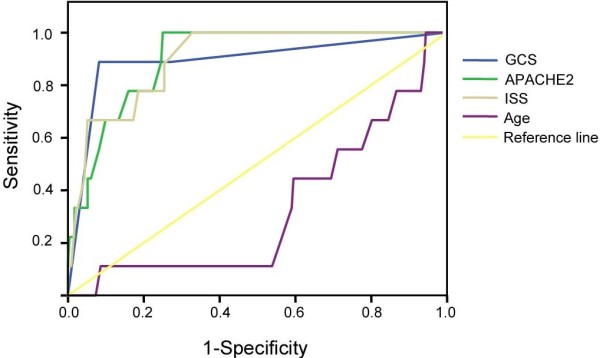
**ROC curve analysis of age, GCS score, ISS score, and APACHE2 score for multiple trauma with lung contusion.**

ROC curve analysis of the prognosis of multiple injuries combined with lung contusion (Figure 1) showed that the area under the ROC curve was 0.330, and the standard error was 0.086, which was not a statistically significant relationship with prognosis (*P* = 0.085). The area under the ROC curve of APACHE2 scores was 0.904, and the standard error was 0.033, which indicated a statistically significant relationship with prognosis (*P* = 0.000). If the APACHE2 score of 16.5 was used as the critical point, the prognosis prediction sensitivity and false positive rates were 0.889 and 0.754 (1-specificity = 1―0.246), respectively. This result suggests that a higher APACHE2 score indicates a greater likelihood of a prognosis of death. When the area of the 95% confidence interval did not include 0.5, the conclusion was the same. The area under the ROC curve of ISS score was 0.902, and the standard error was 0.038. This was a statistically significant relationship with prognosis (*P* = 0.000). If the ISS score was set with 16.5 as the critical point, the prognosis prediction sensitivity and false positive rates were 0.889 and 0.741(1-specificity = 1-0.259), respectively. These results suggest that a higher ISS score indicates a greater likelihood of a prognosis of death. When the area of the 95% confidence interval did not include 0.5, the conclusion was the same. The area under the ROC curve for the GCS score was 0.893, and the standard error was 0.068, which was a statistically significant relationship with prognosis (*P* = 0.000). If the GCS score were set with 5.5 as the critical point, the prognosis prediction sensitivity and false positive rates were 0.919 and 0.889 (1-specificity = 1-0.111), respectively. This result suggests that a higher GCS score indicates a greater likelihood of a prognosis of death. When the area of the 95% confidence interval did not include 0.5, the conclusion was the same.

### Health economic evaluation

The total hospitalization time for those who did not survive was longer than that of those who survived (11.75 ± 10.87d *vs* 50.00 ± 50.07d, *P* = 0.010), and those who died tended to have higher hospitalization costs (98682.63 ± 104360.99RMB *vs* 86883.88 ± 91855.91RMB, *P* = 0.057, Table 3). For the patients with multiple trauma and lung contusion complicated by ARDS, the ICU stay (28.31 ± 51.01d *vs* 8.43 ± 7.93d, *P* = 0.000), total hospitalization time (56.85 ± 59.81d *vs* 43.95 ± 45.30d, *P* = 0.022), and the cost of treatment also increased significantly (152816.46 ± 144291.31 *vs* 66635.67 ± 53894.77, *P* = 0.000, Table 4).

**Table 3:** **Comparison of prognosis and health economics indicators for patients with multiple injuries with lung contusion**

Items	Death group	Survival group	***P-***value
ICU stay (d)	11.50 ± 11.05	13.45 ± 28.15	0.904
Total hospitalization time (d)	11.75 ± 10.87	50.00 ± 50.07	0.010
Hospitalization cost (RMB)	98682.63 ± 104360.99	86883.88 ± 91855.91	0.057

**Table 4:** **Comparison of prognosis and health economics in the patients of multiple trauma and lung contusion with ARDS and non-ARDS**

Items	ARDS group	Non-ARDS groups	***P-***value
ICU stay (d)	28.31 ± 51.01	8.43 ± 7.93	0.000
Total hospitalization time (d)	56.85 ± 59.81	43.95 ± 45.30	0.022
Hospitalization cost (RMB)	152816.46 ± 144291.31	66635.67 ± 53894.77	0.000

## Discussion

Our research found that approximately 35% of patients experienced lung contusion after a multiple trauma incident [9]. Traffic accidents were the most common cause of multiple trauma (64.7%) and were the highest cause (88.9%) of mortality. The average ICU stay of patients with multiple trauma complicated by lung contusion was approximately 13 days, and the total length of stay was approximately 37 days. The cost of hospitalization was approximately 57,000 RMB. The overall mortality rate was 3.58%, which was significantly lower than the rate presented in other reports [9].

Patients with post-traumatic pulmonary contusion could sustain damage to the small airways, alveoli and capillaries, destruction of endothelial cells and epithelial cells, and an increase of pulmonary capillary permeability, which could initiate alveolar edema. They could also experience acute oxygenation deterioration, airway obstruction caused after bronchial blood spilling into the normal tissue, bronchospasm, airway collapse, ventilation/perfusion ratio imbalance, a decrease in lung compliance and effective capacity (24 h after lung contusion reached a peak) [10], pulmonary diffusion dysfunction, hypoxemia, hypoxemia caused by pulmonary vasoconstriction, and increased pulmonary vascular resistance, which results in pulmonary hypertension and pulmonary shunting leading to local over-perfusion, all of which could lead to the aggravation of pulmonary edema and form a vicious cycle [11]. Similarly, the mucus generate increased, the ability of expectoration decreased, and the surfactant generated by injury alveolar tissue was inactivated and led to pulmonary dysfunction [12]. Generally, the damage can be observed on the chest X-ray after 4 to 6 hours; however, these finding are not as obvious on plain flims [13]. Inflammatory reactions gradually subside after 3 to 5 days as the condition eases. Secondary pulmonary dysfunction is generally caused by local inflammatory reactions, blood clots, SIRS, and hospital-acquired pneumonia [14]. Compared with the patients without lung contusion, the patients with lung contusion after multiple trauma had a higher risk of complications (such as pneumonia and ARDS), which may also cause a poor long-term prognosis of respiratory function. However, complications were not an independent risk factor for the death of trauma patients [15]. After admission, such symptomatic treatments as damage control surgery, protective ventilation strategy, fluid resuscitation to maintain stable hemodynamics, and the prevention of infection might mitigate the impact of lung contusion.

Shock is a common symptom of multiple trauma patients admitted to the hospital. If shock is not corrected within gold hour, the risk of death and the risk of complications are significantly higher. A single-factor analysis of risk factors in the study found that the risk of death in patients with lung contusion combined with shock symptoms on admission was significantly higher (χ2 = 15.869, *P* = 0.000). A logistic regression analysis found that shock and poor prognosis trended toward a relationship (*OR* = 9.121, *P* = 0.067) and that the risk of ARDS complications suffered by patients with shock also increased significantly (χ^2^ = 8.214, *P* = 0.004). Tissue hypoperfusion, hypoxemia, ischemia-reperfusion injury, the destruction of intestinal mucosal barrier function, microcirculation dysfunction or failure, cell metabolism disorder, acidosis, changes in cell structure and function, apoptosis, extensive systemic inflammatory response, MODS, and death could be caused by shock [16]. Appropriate and timely fluid resuscitation could quickly add effective circulating volume, improve tissue perfusion, oxygenation, and metabolism, and prevent or mitigate organ dysfunction, thereby improving the prognosis.

Nearly one-third of the severe multiple trauma patients showed varying degrees of coagulation dysfunction during early injury, which presented as endogenous fibrinolytic activity and a reduction of antifibrinolytic protein III, even DIC. The mortality of patients with post-traumatic coagulopathy was significantly higher than that of patients without this coagulopathy [17], which is an independent risk factor for death [18]. This conclusion was consistent with the results of our study (χ^2^ = 14.881, *P* = 0.000). A significantly increased risk of ARDS was associated with coagulation disorders (χ^2^ = 7.459, *P* = 0.006). Traumatic shock is one of the important mechanisms that cause coagulation disorders. Lung micro-vascular injury causes the release of tissue factor, activation of the extrinsic coagulation pathway, deposition of fibrinogen, platelet activation, and the release of pro-inflammatory mediators. Then, it induces the formation of micro-vascular thrombi, thereby further deteriorating tissue damage and the consumption of clotting factors, which leads to bleeding, injury, and hypoxemia. Tissue hypoperfusion activates endothelial cells and the protein C pathway.

This conclusion was consistent with the increased risk of morality with gastrointestinal bleeding that was found in our study. Gastrointestinal bleeding occurred more frequently in critically ill patients in the ICU and only influenced the prognosis and disease if the onset occurred within 24 hours. The risk coefficient decreased over time, which may be due to early routine acid suppression therapy, gastric mucosal protective therapy, and improvement of gastrointestinal hypo-perfusion due to improving hypoxemia and hypotension. Only 4 patients in the study experienced gastrointestinal bleeding, and there was a positive significant relationship with prognosis (χ^2^ = 5.398, *P* = 0.02).

Early blood transfusion is considered to be an important reason for infusion-related deaths. Our study confirmed that the likelihood of mortality and ARDS complications was significantly higher in those with lung contusion compared with non-early transfusion patients (*P* = 0.006, *P* = 0.000). It is possible that the transfusion activated neutrophils, released medium that induced lung injury, aggravated the deterioration of the patient's oxygenation, and influenced the prognosis of patients.

Pre-hospital care for trauma patients, such as mechanical ventilation for patients with severe respiratory dysfunction, could improve the patient's respiratory function, open the alveolar and collapsing airway, increase lung volume, improve ventilation/perfusion, improve lung compliance, and correct tissue hypoxia. However, our study found that the risk of death and ARDS was greatly increased in patients who received mechanical ventilation. Hypoxemia and hypoperfusion, which was difficult to correct, could possibly lead to tissue and organ dysfunction and could increase the risk of death and complications.

Only 5.1% of the patients with multiple trauma and pulmonary contusion required surgery, the need for which was mainly determined by the condition of the chest, the severity of pulmonary hemorrhage, or the degree of damage to the other organs [19]. The increased risk of death in patients with emergency surgery might be because of increased trauma severity, as well as the risks of the surgery itself.

There was little impact on the prognosis of patients with multiple trauma and lung contusion when admitted to the hospital (χ^2^ = 0.63, *P* = 0.427), but an increased risk of ARDS complications was observed (χ^2^ = 8.058, *P* = 0.005). In China, the use of antibiotics for multiple trauma is standard without considertion of co-infection. Therefore, the use of antibiotics was considered as a constant and was not analyzed as a risk factor of prognosis and ARDS.

The results of our study found that the use of hormone did not increase the risk of poor prognosis (*P* = 0.16), but it did significantly increase the risk of ARDS (*P* = 0.002). These results are consistent with the clinical phenomenon that we observed, which demonstrated that the use of the hormone does not improve the prognosis but only affects the condition and symptoms. We considered that the hormone could not only treat pulmonary contusion but also prevent the aggravation of the patient's lung contusion and shock and shorten the time of the anti-shock. Early, short-range, high-dose corticosteroids could reduce the injury to alveolar epithelial and capillary endothelial cells, reduce the non-specific inflammation, reduce pulmonary vascular permeability, effectively improve microcirculation, shorten the inflammatory reaction time, and effectively reduce pulmonary edema [20]. An animal model also showed that the use of dexamethasone after 4 hours of trauma resuscitation could significantly reduce the plasma concentration of endotoxin, improve the mean arterial blood pressure, and improve the tissue oxygen tension of the liver and small intestine. The effect of large doses of hormones was more significant [21]. A study by Liu et al. confirmed that the early use of methylprednisolone in the treatment of trauma with pulmonary contusion significantly reduced the inflammatory reaction within 24 hours compared with the control group. The PO_2_/FiO_2_ values (127.18 ± 8.39) of those who received steroids were higher than those of the control group (*P* < 0.05). The experimental results demonstrated that the early use of steriod could improve long-term prognosis, shorten the course, and greatly reduce mortality (*P* < 0.01). CT and chest X-ray observations also confirmed that the methylprednisolone therapy group had a higher absorption rate of the lung tissue fluid and recovery of organizational structure [22]. The possible mechanism for this was that the anti-inflammatory effects mediated by glucocorticoid receptors could stabilize the lysosomal membrane, reduce pulmonary vascular permeability, reduce pulmonary leukocyte exudate and alveolar membrane edema, improve the alveolar ventilation/perfusion ratio, control macrophage phagocytosis of the antigen, improve SIRS, reduce pulmonary vascular resistance, and correct shock. Its diuretic effect could reduce excess water and sodium and therefore relieve pulmonary vascular resistance and reduce the burden on the right side of the heart [23].

The APACHE2, GCS, and ISS scores are reliable prognostic indicators for multiple trauma patients and were closely related to the occurrence of ARDS [4]. This result was consistent with the findings of our study. In our study, if the GCS < 5.5 (sensitivity = 0.919, specificity = 0.889), the APACHE2 score > 16.5 (sensitivity = 0.889, specificity = 0.754), or the ISS score > 20.5 (sensitivity = 0.889, specificity = 0.741), the risk of death was significantly elevated. The total hospitalization time of patients who died was significantly longer, and these patients had higher hospitalization costs and an increased risk of ARDS.

After trauma, the incidence of ARDS and other complications for patients with pulmonary contusion was 35% and the hospitalization costs were far greater than those of the patients without complications ($59,633 *vs* $24,715) [21]. In our study, only 10.32% (26 cases) of patients’ conditions were complicated by ARDS, which is significantly lower than the reported rates of 31% to 74%. Logistic regression analysis found that pre-hospital intubation (OR = 18.633, *P* = 0.000) and drainage (*OR* = 2.911, *P* = 0.029) were independent risk factors for ARDS. This indirectly showed that the occurrence of ARDS was closely related to the control of lung damage and respiratory function. For patients with lung contusion and multiple injuries, pre-hospital intubation could quickly and effectively open airways and improve breathing function and could clear airway secretions to prevent aspiration. Drainage was the main method for controlling hemothorax or hemopneumothorax. Patients with ARDS had a significantly increased length of stay in the ICU (28.31 ± 51.01d *vs* 8.43 ± 7.93d), total hospitalization time (56.85 ± 59.81d *vs* 43.95 ± 45.30d), hospital spending (152816.46 ± 144291.31 yuan *vs* 66635.67 ± 53894.77 yuan), and risk of death (*P* < 0.05). The results indicated that actively preventing the occurrence of ARDS may not only have a positive impact on the patient's prognosis but could also significantly reduce hospital costs, shorten hospital stays, and reduce the use of hospital resources, which plays an active role in the social effects and health economics.

Our study has some limitations. It was a single-center study and could not avoid the inclusion of disease, which was not widely representative. Retrospective study designs have universal disadvantages; the data of clinical cases span years, and there are likely to be misdiagnoses and incomplete medical records. Finally, the study did not rule out pre-trauma cases combined with other pathological factors. The number of deaths and cases of ARDS were small in number, all of which could lead to a deviation of results.

Admission to hospital with shock, coagulopathy, need for emergency surgery, pre-hospital intubation, and mechanical ventilation could significantly increase the risk of mortality and ARDS incidence for patients with multiple trauma and pulmonary contusion. Patients with these factors would have a poor prognosis with gastrointestinal bleeding. A high ISS score, high APACHE2 score, and low GCS score were independent risk factors for poor prognosis. If patients had an infection, had a chest tube placed, or received hormones and suctioning, there was a higher risk of ARDS; pre-hospital intubation and drainage were independent risk factors of ARDS. In patients with ARDS, the ICU stay, total length of stay, and hospital costs might increase significantly. A GCS score < 5.5, APACHE2 score > 16.5 and ISS score > 20.5 could be considered indicators of poor prognosis for patients with multiple trauma and lung contusion.

## Conclusion

Those patients with multiple trauma and pulmonary contusion admitted to the hospital with shock, coagulopathy, a need for emergency surgery, pre-hospital intubation, and a need for mechanical ventilation could have a significantly increased risk of mortality and ARDS incidence. A risk for poor prognosis was associated with gastrointestinal bleeding. A high ISS score, high APACHE2, and low GCS score were independent risk factors for poor prognosis.

## Abbreviations

ALI: Acute lung injury
ARDS: Acute respiratory distress syndrome
VAP: Ventilator associated pneumonia
MODS: Multiple organ dysfunction syndrome
APACH: Acute physiology and chronic health evaluation score system
ISS: Injury severity score.
